# Hyaluronic acid concentration in liver diseases

**DOI:** 10.1007/s10238-015-0388-8

**Published:** 2015-09-09

**Authors:** Monika Gudowska, Ewa Gruszewska, Anatol Panasiuk, Bogdan Cylwik, Robert Flisiak, Magdalena Świderska, Maciej Szmitkowski, Lech Chrostek

**Affiliations:** 1 Department of Biochemical Diagnostics, Medical University of Bialystok, Waszyngtona 15A St., 15-269 Białystok, Poland; 2 Department of Infectious Diseases and Hepatology, Medical University of Bialystok, Zurawia 14 St., 15-540 Białystok, Poland; 3 Department of Pediatric Laboratory Diagnostics, Medical University of Bialystok, Waszyngtona 17 St., 15-269 Białystok, Poland

**Keywords:** Hyaluronic acid, Liver fibrosis, Liver cirrhosis, Toxic hepatitis

## Abstract

The aim of this study was to evaluate the effect of liver diseases of different etiologies and clinical severity of liver cirrhosis on the serum level of hyaluronic acid. The results were compared with noninvasive markers of liver fibrosis: APRI, GAPRI, HAPRI, FIB-4 and Forn’s index. Serum samples were obtained from 20 healthy volunteers and patients suffering from alcoholic cirrhosis (AC)—57 patients, non-alcoholic cirrhosis (NAC)—30 and toxic hepatitis (HT)—22. Cirrhotic patients were classified according to Child–Pugh score. Hyaluronic acid concentration was measured by the immunochemical method. Non-patented indicators were calculated using special formulas. The mean serum hyaluronic acid concentration was significantly higher in AC, NAC and HT group in comparison with the control group. There were significant differences in the serum hyaluronic acid levels between liver diseases, and in AC they were significantly higher than those in NAC and HT group. The serum hyaluronic acid level differs significantly due to the severity of cirrhosis and was the highest in Child–Pugh class C. The sensitivity, specificity, accuracy, positive and negative predictive values and the area under the ROC curve for hyaluronic acid and all non-patented algorithms were high and similar to each other. We conclude that the concentration of hyaluronic acid changes in liver diseases and is affected by the severity of liver cirrhosis. Serum hyaluronic acid should be considered as a good marker for noninvasive diagnosis of liver damage, but the combination of markers is more useful.

## Introduction

The common pathway leading to liver fibrosis and cirrhosis is growing deposition of extracellular matrix (ECM) [1]. It results from molecular and histological rearrangement of collagens, glycoproteins and hyaluronans [2]. The main structural role in the formation of ECM plays hyaluronic acid (HA) [1, 2]. This polysaccharide is high molecular glycosaminoglycan that is practically found in every tissue in the body, and is synthesized in synovial lining cells and hepatic stellate cells (HSC) by an enzyme called hyaluronic acid synthases [3, 4]. In the liver, HA is degraded by the sinusoidal endothelial cells [3, 4]. Owing to hepatic metabolism, the serum HA levels can be affected by liver cells injury and by transformation of stellate cells to myofibroblasts induced by inflammatory reactions [5]. Because of that and its short half-life in blood (2–5 min), serum HA levels can reflect liver fibrosis stage and were incorporated into a long list of serum noninvasive liver fibrosis markers such as procollagen III N-terminal propeptide (PIIINP), laminin and transforming growth factor beta (TGF-β) [8]. It was found that serum levels of hyaluronic acid are elevated in chronic liver diseases in which the serum levels of ECM would be changed. These include alcoholic and non-alcoholic steatohepatitis, hepatitis B, C and others [5, 6]. In these clinical conditions HA might be used alone or in an algorithm models that use HA as a major constituent. Some of them, i.e., HAPRI, involve combinations of direct and indirect markers of hepatic fibrosis.

The aim of this study was to evaluate the effect of liver diseases of different etiologies (alcoholic and non-alcoholic) and clinical severity of liver cirrhosis on the serum level of hyaluronic acid. The results were compared with non-patented, noninvasive indicators of liver fibrosis: APRI, GAPRI, HAPRI, FIB-4 and Forn’s index.

## Materials and methods

### Subjects

The experimental group consisted of 109 patients consecutively admitted to the Department of Infectious Diseases and Hepatology (Medical University of Bialystok). The patients tested were males (74) and females (35) (age range 26–88 years). They were divided into three subgroups according to the clinical diagnosis of disease: alcoholic cirrhosis (AC)—57 patients, non-alcoholic cirrhosis (NAC)—30 patients and toxic hepatitis (HT)—22 patients. Non-alcoholic cirrhosis was caused by chronic hepatitis C—13 patients, chronic hepatitis B—1, autoimmune hepatitis—1, primary biliary cirrhosis—4 and undefined factors—11. The severity of liver cirrhosis was evaluated by Child–Pugh score (class A—27, class B—31 and class C—25). The diagnosis was based on the clinical data [signs, symptoms, physical exams, biochemical liver panel, abdominal ultrasound or computed tomography (CT) scan of the abdomen] and the liver biopsy only in selected cases. To confirm the diagnosis of HCV, anti-HCV test was performed. All patients were interviewed regarding their use of alcohol. The acute alcohol abuse was the cause of seven cases of toxic hepatitis. Control group consisted of 20 healthy volunteers (11 males and 9 females) recruited from hospital workers.

Informed consent was obtained from all individual participants (healthy and sick) included in the study. This study was in accordance with Helsinki declaration and was approved by the Bioethical Committee at the Medical University in Bialystok.

### Blood sampling

Blood fasting samples were taken by vein puncture after admittance and before treatment. The sera were separated by centrifugation and stored at −86 °C until assayed. Besides serum, a part of each blood sample was collected into tubes containing 3.8 % liquid sodium citrate for hemostasis analyses hemostasis and EDTA-2 for hematological analyses.

Hyaluronic acid (HA) was measured on the Architect ci8200 (Abbott Laboratories, Abbott Park, IL, USA) according to the immunochemical method using WAKO reagents. Prothrombin time (PT) was determined on STA Compact Max analyzer (Diagnostica Stago, France) by viscometric method. PLT count was determined on Sysmex XS-800i (Sysmex Corporation, Singapore).

### Calculations

APRI, GAPRI, HAPRI, Fib-4 and Forn’s index were calculated on the basis of following formulas:

$$\begin{aligned} {\text{APRI}} & = (({\text{AST}}\,[{\text{IU}}/{\text{L}}]/50\,{\text{IU}}/{\text{L)}}/{\text{PLT}}\, [10^{9} /{\text{L]}}) \times 100 \\ {\text{GAPRI}} & = ({\text{GGT}}\,[{\text{IU}}/{\text{L}}]/{\text{PLT}}\,[10^{9} /{\text{L}}]) \times 100 \\ {\text{HAPRI}} & = ({\text{HA}}\, [ {\text{ng/mL]/INR}}) \times 100 \\ {\text{Fib-}}4 & = ({\text{age}} \times {\text{AST}}\, [ {\text{IU}}/{\text{L]}})/({\text{PLT}}\,[10^{9} /{\text{L}}]) \times \surd (({\text{ALT}}\,[{\text{IU}}/{\text{L}}])) \\ {\text{Forn}\text{'} \text{s index}} & = 7.811 - 3.131\,{\text{ln (PLT}}\, [10^{9} /{\text{L])}} + \, 0.781\,{\text{ln (GGT}}\, [ {\text{IU}}/{\text{L])}} \\ & \quad \quad + \,3.467\,{\text{ln (age)}} - 0.014\,({\text{cholesterol}}\, [ {\text{mg/dL]}}) \\ \end{aligned}$$

### Statistical analysis

The differences between tested and control groups were evaluated by Mann–Whitney *U* test. To test the hypothesis about the differences between liver diseases, ANOVA rank Kruskal–Wallis test was performed. To calculate the correlation between variables, Spearman’s rank correlation coefficient was used. We considered *P* values <0.05 as statistically significant. The diagnostic performance of each test was calculated as sensitivity, specificity, PPV, NPV and accuracy. The diagnostic values of the tests were compared by the area under the receiver operating characteristic (AUROC) curve.

## Results

The changes in serum hyaluronic acid and noninvasive markers are presented in Table 1. In all liver diseases, the serum hyaluronic acid concentration was higher in comparison with the control group (*P* = 0.000 for all comparisons). Also, the values of all noninvasive hepatic fibrosis indicators: APRI, GAPRI, HAPRI, Forn’s index and FIB-4, were increased when compared to the controls in AC, NAC and HT (*P* < 0.001 for all comparisons). Liver diseases affect the serum HA concentrations (ANOVA rank Kruskal–Wallis test: *H* = 27.17; *P* < 0.001). The patients with alcoholic cirrhosis had higher serum HA concentration compared with non-alcoholic cirrhosis and toxic hepatitis (*P* = 0.009 and *P* < 0.001, respectively), but there were no significant differences between non-alcoholic cirrhosis and toxic hepatitis (*P* = 0.118). The Spearman’s rank correlation test demonstrated an association between degree of liver damage in Child–Pugh scale and hyaluronic acid concentration (*R* = 0.679, *P* = 0.000). ANOVA rank Kruskal–Wallis test revealed that in class C, hyaluronic acid level was higher than that in classes B and A (*P* = 0.002 and *P* < 0.001, respectively). Additionally, hyaluronic acid concentration was higher in class B than that in class A (*P* = 0.007).

**Table 1:** Results of laboratory tests in controls (C) and patients with liver diseases and according to Child–Pugh score

Group	HA (ng/L)	APRI	GAPRI	HAPRI	Forn’s index	FIB-4
Controls (*n* = 20)	0.03 ± 0.01	0.21 ± 0.06	10.42 ± 4.12	3677.82 ± 1032.71	1.98 ± 1.03	0.75 ± 0.30
AC (*n* = 57)	1.08 ± 1.13*^b,c^	3.14 ± 4.33*	479.53 ± 1020.76*^b^	71922 ± 68232.26*^b,c^	8.94 ± 3.21*^c^	12.27 ± 16.10*^c^
NAC (*n* = 30)	0.40 ± 0.35*^a^	2.75 ± 5.62*	152.63 ± 191.95*^a,c^	34009.15 ± 27452.93*^a^	8.94 ± 1.86*^c^	7.13 ± 5.29*^c^
HT (*n* = 22)	0.19 ± 0.18*^a^	1.75 ± 1.97*	639.48 ± 976.10*^b^	19696.46 ± 20834.80*^a^	6.24 ± 2.10*^a,b^	3.59 ± 4.61*^a,b^
Child–Pugh A (*n* = 27)	0.31 ± 0.32*^B,C^	2.63 ± 6.21*^C^	459.48 ± 1313.81*	28605.83 ± 29820.71*^B,C^	8.09 ± 3.31*^C^	6.98 ± 15.22*^C^
Child–Pugh B (*n* = 31)	0.87 ± 0.95*^A,C^	3.11 ± 4.73*^C^	490.19 ± 1240.27*	68943.70 ± 84528.97*^A^	8.47 ± 2.41*^C^	10.23 ± 14.50*^C^
Child–Pugh C (*n* = 25)	2.22 ± 2.40*^A,B^	4.50 ± 5.00*^A,B^	666.1 ± 1405.40*	125975.00 ± 11295.5*^A^	10.80 ± 1.60*^A,B^	17.30 ± 17.10*^A,B^

Data are mean ± standard deviation

*AC* alcoholic cirrhosis, *NAC* non-alcoholic cirrhosis, *HT* toxic hepatitis

The significant differences in comparison with (ANOVA rank Kruskal–Wallis): ^a^AC; ^b^NAC; ^c^HT; ^A^Child–Pugh A; ^B^Child–Pugh B; ^C^Child–Pugh C

* *P* < 0.05 the differences between tested group and controls (Mann–Whitney *U* test)

The values of all noninvasive hepatic fibrosis indexes: APRI, GAPRI, HAPRI, Forn’s index and FIB-4, were increased when compared to the controls in AC, NAC and HT group (*P* < 0.001 for all comparisons). The analysis of variance revealed that liver diseases effect the scores of GAPRI, HAPRI, Forn’s index and FIB-4 (*H* = 11.488, *P* = 0.005; *H* = 21.490, *P* < 0.001; *H* = 18.668, *P* < 0.001; and *H* = 18.985, *P* < 0.001, respectively). Post hoc analysis showed that values of HAPRI, Forn’s index and FIB-4 were increased in AC group in comparison with HT group (*P* < 0.001 for both). Scores of Forn’s index and FIB-4 were also higher in NAC group than those in HT (*P* < 0.001 for all comparisons). The analysis of GAPRI score revealed that it was significantly lower in NAC than that in HT and AC (*P* = 0.010 for both). HAPRI score was higher in AC group in comparison with NAC (*P* = 0.030). ANOVA rank Kruskal–Wallis analysis showed that Child–Pugh stage effects on the APRI, HAPRI, Forn’s index and FIB-4 (*P* < 0.05 for all comparisons). Further analysis showed that theirs values were higher in class C than those in class A (*P* < 0.05 for all comparisons). Additionally, APRI, HAPRI, Forns’s index and FIB-4 were higher in Child–Pugh class C those that in class B (*P* = 0.004, *P* = 0.014, *P* < 0.001 and *P* < 0.001, respectively). HAPRI level was also increased in class A compared with class B (*P* < 0.05).

Diagnostic usefulness of hyaluronic acid, APRI, GAPRI, HAPRI, FIB-4 and Forn’s index in liver diseases is presented in Table 2. Hyaluronic acid and GAPRI had the highest and the same ability to detect (98.2 % of sensitivity for both) and exclude (with 100 % specificity for both) of alcoholic cirrhosis. Additionally, our study showed that Forn’s index and GAPRI with 100 % sensitivity correctly identify all patients with the non-alcoholic cirrhosis. In case of ability to exclude non-alcoholic cirrhosis, four indicators had 100 % specificity: Forn’s index, APRI, FIB-4 and HAPRI (in order dependent on the NPV). The highest sensitivity and PPV in detection of toxic hepatitis have GAPRI. GAPRI, APRI, HA and HAPRI with 100 % specificity correctly identify all patients without toxic hepatitis.

**Table 2:** Diagnostic value of hyaluronic acid, APRI, GAPRI, HAPRI, FIB-4 and Forn’s index in liver diseases

Liver disease	Cutoff (from ROC)	Sensitivity (%)	Specificity (%)	ACC (%)	PPV (%)	NPV (%)	AUC ± SE
**Hyaluronic acid**							
AC	0.55 ng/mL	98.2	100.0	98.7	100.0	95.2	0.996 ± 0.005
NAC	0.72 ng/mL	93.3	95.0	96.0	100.0	90.9	0.988 ± 0.011
HT	0.62 ng/mL	77.3	100.0	88.1	100.0	80.0	0.884 ± 0.062
**APRI**							
AC	0.27	92.7	85.0	0.907	94.4	81.0	0.936 ± 0.029
NAC	0.34	96.4	100.0	0.979	100.0	95.2	0.996 ± 0.005
HT	0.43	81.00	100.0	0.902	100.0	83.3	0.948 ± 0.032
**GAPRI**							
AC	29.83	98.2	100.0	0.987	100.0	95.2	0.998 ± 0.002
NAC	17.722	100.0	90.0	0.959	93.5	100.0	0.993 ± 0.007
HT	25.10	100.0	100.0	1.0	100.0	100.0	1.0 ± 0.0
**HAPRI**							
AC	6453.49	96.5	100.0	0.974	100.0	90.9	0.993 ± 0.007
NAC	8148.15	93.3	100.0	0.960	100.0	90.9	0.97 ± 0.012
HT	6126.13	77.3	100.0	0.881	100.0	80.0	0.875 ± 0.064
**FIB-4**							
AC	1.51	94.4	100.0	0.959	100.0	87.0	0.975 ± 0.019
NAC	1.66	96.3	100.0	0.979	100.0	95.2	0.993 ± 0.009
HT	1.37	81.0	95.0	0.878	94.4	82.6	0.933 ± 0.039
**Forn's index**							
AC	4.60	94.5	100.0	0.960	100.0	87.0	0.967 ± 0.022
NAC	4.44	100.0	100.0	1.0	100.0	100.0	1.0 ± 0.0
HT	3.21	90.5	95.0	0.927	95.0	90.5	0.955 ± 0.033

*AC* alcoholic cirrhosis, *NAC* non-alcoholic cirrhosis, *HT* toxic hepatitis, *ACC* diagnostic accuracy, *PPV* positive predictive value, *NPV* negative predictive value, *AUC* area under ROC curve, *SE* standard error

The highest diagnostic power (AUC) in detection of liver diseases has GAPRI (mean ± SE; 0.997 ± 0.022). All the other indicators: Forn’s index, FIB-4, APRI, HA and HAPRI had smaller mean value of AUC (0.974 ± 0.018, 0.967 ± 0.022, 0.960 ± 0.022, 0.956 ± 0.026 and 0.946 ± 0.083, respectively). In case of all of them, it was an excellent diagnostic power.

## Discussion

Liver biopsy still remains a “gold standard” for diagnosing liver disease, but serum markers might be useful in patients in whom a liver biopsy is not recommended or would be associated with increased risk of complications. Problematic are also situations in which an access to histopathology experts is limited and when the result is required immediately. Therefore, we tried to examine the diagnostic value of simple fibrosis indicator such as hyaluronic acid in patients with liver diseases and compare it to markers closed in algorithm model.

We have shown that the mean serum level of hyaluronic acid is elevated in patients with liver injury. It is well known that the changes in hyaluronic acid concentration in liver disease may originate from increased liver fibrogenesis and fibrolysis [10]. Both of them may cause increased level of circulating ECM component or their fragments. Due to the fact that hyaluronic acid plays the main structural role in the ECM formation, its concentration in the sera rises rapidly in case of liver damage [10]. Some studies showed that hyaluronic acid is not able to differentiate cirrhotic and non-cirrhotic conditions [9], but our studies have shown that the levels of serum HA concentration differ between cirrhosis of alcoholic origin and toxic hepatitis and were higher in cirrhosis. There was also difference between alcoholic and non-alcoholic cirrhosis. Studies based on hyaluronic acid as an liver damage indicator suggest that the mechanism of hyaluronic acid growth is slightly different in inflammation (such a toxic hepatitis) and non-alcoholic cirrhosis than in the mechanisms involved in alcoholics [10, 11]. Rockey and Montgomery [10] suggest that non-alcoholic liver diseases and inflammation provide to activation of HSC, which undergoing morphological and functional changes. Among the functional changes, the most important is transformation of HSC to myofibroblasts and effective secretion of ECM proteins to the blood and biological fluids [5, 10]. The excess ECM including hyaluronic acid produced after liver injury is taken down when the repair process is completed. It is important to take into account that an advanced cirrhosis is a result of long-lasting process of chronic injury and fibrosis, where repair processes are defective. Therefore, HA concentrations in cirrhosis might be higher than those in toxic hepatitis [10]. Moreover, in 15–40 % patients with hepatic inflammation and fibrosis may develop irreversible liver cirrhosis and hepatocellular carcinoma. Naveau et al. studied the associations between hyaluronic acid concentration and stage of chronic alcohol liver disease. Similar to our study, the concentration of investigated polysaccharide was higher in patients with alcoholic hepatitis (as an example of toxic hepatitis) than without, but lower than for stages F3 and F4 (cirrhosis). Authors also compared diagnostic power of HA with FibroTest (including five biochemical markers, corrected for sex and weight). The AUCs for >F2 stages were lower for hyaluronic acid, but for F2–F4, the differences were not significantly [18].

We speculated that very high hyaluronic acid concentrations in alcoholic cirrhosis in our and Naveau et al.’s studies are probably caused by a combination of increased ECM production and two extra ways. These two ways have been identified and described on mice and rats models. Firstly, researchers have shown that alcohol effects of hyaluronic acid by modifications of communication between liver cells affect HA clearance [12]. Secondarily, alcohol may lead to dysfunction of sinusoidal endothelial cells responsible for degradation of ECM excess [12, 13]. It is most likely that a combination of all these factors resulted in tremendous growth of hyaluronic acid concentration in patients with alcoholic cirrhosis.

The correlation study demonstrated an association between severity of liver cirrhosis (Child–Pugh scale) and serum hyaluronic acid concentrations. These results showed that the HA concentrations were the highest in the most severe stage of liver injury (Child–Pugh class C). We denoted about 2.5-fold of mean HA level in class C in comparison with class B and sevenfold in comparison with class A. The difference might be associated with long-lasting chronic liver injury and fibrosis, and growing production of ECM and dwindling hepatic clearance [5, 7, 14]. There are no other studies comparing hyaluronic acid concentration with severity of liver damage expressed in Child–Pugh scale. We have shown that HA concentration was only compared with various stages and grading of alcoholic liver diseases (ALD). In the study of Stickel et al. [17], the authors demonstrated an elevated HA level in patients with ALD and correlation with degree of alcohol-related liver damage. Serum HA increased with severity of liver damage from fatty liver through fatty liver and fibrosis, fatty liver and inflammation, severe fibrosis and inflammation to cirrhosis with the highest of HA levels. These studies confirm that increase in HA concentration is associated with dysfunction of sinusoidal endothelial cell and increased synthesis by HSC and fibroblasts.

Our study showed that hyaluronic acid has the highest diagnostic sensitivity, ACC and NPV in alcoholic cirrhosis. Diagnostic specificity was the same in alcoholic cirrhosis and toxic hepatitis—each a 100 %. Additionally, hyaluronic acid has 100 % PPV in all liver diseases. It is a consequence of the lack of false negative results. Valva et al. [15] compared a single marker (hyaluronic acid, TIMP-1 and PIIINP) with panel combined with hyaluronic acid, named ELF test including HA, TIMP-1 and PIIINP. They showed that the combination of these markers is more credible to evaluate the degree of liver fibrosis in HCV patients than each marker alone. Despite the fact that hyaluronic acid is probably the best individual test reflecting the degree of hepatic fibrosis [16], we tried to compare its diagnostic power with other noninvasive algorithms. Our study revealed that in alcoholic cirrhosis AUC of hyaluronic acid was the highest, just after GAPRI index. On the other hand, in case of non-alcoholic cirrhosis and toxic hepatitis, AUCs of hyaluronic acid and HAPRI index (included HA) were similar, but lower in comparison with another multiple markers: Forn’s, GAPRI, APRI and FIB-4.

A major conclusion is that the concentrations of serum hyaluronic acid are elevated in liver diseases, but different. Additionally, hyaluronic acid levels rise continuously with severity of liver damage expressed in Child–Pugh score. Therefore, hyaluronic acid should be considered as a good marker for noninvasive diagnosis of liver damage. Our study revealed that the combination of markers is more useful than a single marker such a hyaluronic acid.
